# Supplementary material for: Efficacy of a Smartphone Application to Promote Maternal Influenza Vaccination: A Randomized Controlled Trial
Source: Vaccines (Basel). 2022 Feb 27;10(3):369. doi: 10.3390/vaccines10030369 (PMC8954751; doi:10.3390/vaccines10030369)
Supplement: Supplementary file 1 [file vaccines-10-00369-s001.zip › vaccines-1591699-supplementary.pdf]

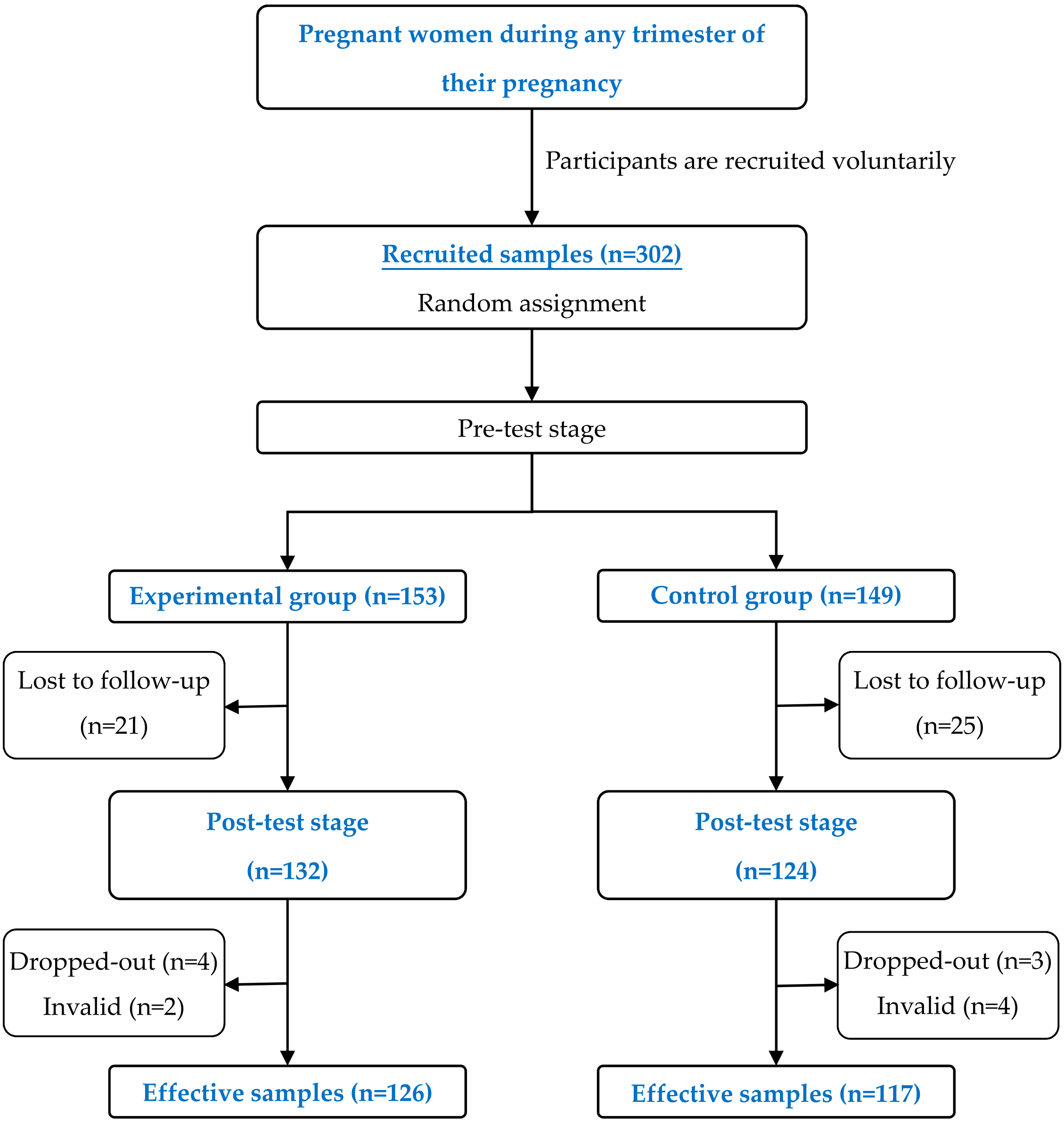

Figure S1. The recruitment of participants and the process of sample collection.

Table S1. The pre- and post-test knowledge scale scores about influenza and its vaccine between two groups of pregnant women.

Knowledge scale about influenza and its vaccine	All (n=243)	Experimental group (n=126)						Control group (n=117)					
	Pre-test	Pre-test		Post-test		Paired-		Pre-tes		Post-test		Paired-	
	CAR[a]	Mean	SD[b]	Mean	SD[b]	t(125)[c]	p	Mean	SD[b]	Mean	SD[b]	t(116)[c]	p
**Total knowledge scale score**	**62%**	**14.00**	**3.41**	**15.63**	**3.75**	**-5.51**	**<.01**	**13.12**	**4.04**	**14.09**	**4.04**	**-4.33**	**<.01**
Characteristics of influenza virus	67%	2.02	0.59	2.13	0.69	-2.17	**.03**	2.03	0.62	2.09	0.58	-1.71	.09
Severity of influenza	61%	3.21	1.23	3.63	1.34	-3.57	**<.01**	2.92	1.40	3.16	1.47	-2.45	**.02**
Benefits of influenza vaccination	65%	4.03	1.15	4.25	1.10	-2.07	**.04**	3.71	1.38	3.97	1.25	-2.90	**.01**
Timing of influenza vaccination	66%	3.37	1.14	3.79	1.08	-4.24	**<.01**	3.24	1.32	3.55	1.22	-4.45	**<.01**
Safety of influenza vaccination	43%	1.38	0.97	1.82	0.94	-5.30	**<.01**	1.22	0.97	1.33	1.00	-1.80	.07

[a] Correct answer rate: Participants who provided the correct answer as a percentage of the total number of participants.
[b] Standard deviation.
[c] Paired samples t-test compared between pre- and post-test scale scores within each of the experimental and control groups.

Table S2. The pre- and post-test attitudes scale scores towards maternal influenza vaccination between two groups of pregnant women.

Attitude scale towards maternal influenza vaccination	All (n=243)		Experimental group (n=126)						Control group (n=117)					
	Pre-test		Pre-test		Post-test		Paired-		Pre-tes		Post-test		Paired-	
	Mean	SD[a]	Mean	SD[a]	Mean	SD[a]	t(125)[b]	p	Mean	SD[a]	Mean	SD[a]	t(116)[b]	p
**Total attitude scale score**	**99.88**	**12.99**	**99.40**	**12.63**	**104.75**	**12.80**	**-5.45**	**<.01**	**100.38**	**13.39**	**101.83**	**13.93**	**-2.20**	**.03**
Perceived susceptibility	17.09	3.06	17.12	3.11	17.89	2.98	-1.36	.18	17.06	3.02	17.42	3.18	-2.46	**.02**
Perceived severity	18.74	2.77	18.80	2.89	19.28	2.73	-1.08	.28	18.67	2.65	19.15	2.62	-3.54	**<.01**
Perceived benefits of action	16.69	3.31	16.50	3.43	17.80	3.16	-4.76	**<.01**	16.90	3.19	17.20	3.49	-1.57	.12
Perceived barriers of action	17.00	3.79	16.90	3.85	17.48	3.50	-2.33	**.02**	17.11	3.73	17.29	3.62	-1.01	.32
Cues to action	15.60	3.12	15.29	2.88	16.21	3.04	-4.23	**<.01**	15.93	3.34	16.04	3.23	-.58	.56
Self-efficacy	14.76	3.21	14.79	3.09	16.10	3.31	-5.22	**<.01**	14.72	3.35	14.73	3.40	-.05	.96

[a] Standard deviation.
[b] Paired samples t-test compared between pre- and post-test scale scores within each of the experimental and control groups.
